# Berberine modulates hyper-inflammation in mouse macrophages stimulated with polyinosinic-polycytidylic acid via calcium-CHOP/STAT pathway

**DOI:** 10.1038/s41598-021-90752-z

**Published:** 2021-05-28

**Authors:** Hyun-Ju Kim, Young-Jin Kim, Wansu Park

**Affiliations:** Department of Pathology, College of Korean Medicine, Gachon University, Seong-nam, 13120 Republic of Korea

**Keywords:** Drug discovery, Immunology, Pathogenesis

## Abstract

Berberine is a well-known quaternary ammonium salt that is usually found in the roots of such plants as *Phellodendron amurense* and *Coptis chinensis*. However, the effects of berberine on double-stranded RNA (dsRNA)-induced macrophages have not been fully reported. In this study, we examined the anti-inflammatory effects of berberine on dsRNA [polyinosinic-polycytidylic acid; poly I:C]-induced macrophages. Levels of nitric oxide (NO), Prostaglandin E2 (PGE2), first apoptosis signal receptor (Fas; CD95), cytokines, intracellular calcium, phosphorylated I-kappa-B-alpha (IkB-α), phosphorylated p38 mitogen-activated protein kinase (MAPK), phosphorylated ERK1/2, phosphorylated signal transducer and activated transcription 3 (STAT3), and mRNA expression of inflammatory genes in poly I:C-induced RAW 264.7 mouse macrophages were evaluated. Berberine significantly inhibited the production of NO, PGE2, Fas, GM-CSF, LIF, LIX, RANTES, and MIP-2 as well as calcium release in poly I:C-induced RAW 264.7 cells at concentrations of up to 50 μM. Berberine also significantly inhibited the phosphorylation of p38 MAPK, ERK1/2, IkB-α, and STAT3 in poly I:C-induced RAW 264.7 cells. Additionally, berberine significantly decreased the mRNA expressions of *Chop* (GADD153), *Stat1*, *Stat3*, and *Fas* in poly I:C-induced RAW 264.7 cells. Taken together, berberine has anti-inflammatory properties related to its inhibition of NO, PGE2, Fas, GM-CSF, LIF, LIX, RANTES, and MIP-2 in dsRNA-induced macrophages via the endoplasmic reticulum stress-related calcium-CHOP/STAT pathway.

## Introduction

**Figure 1:**
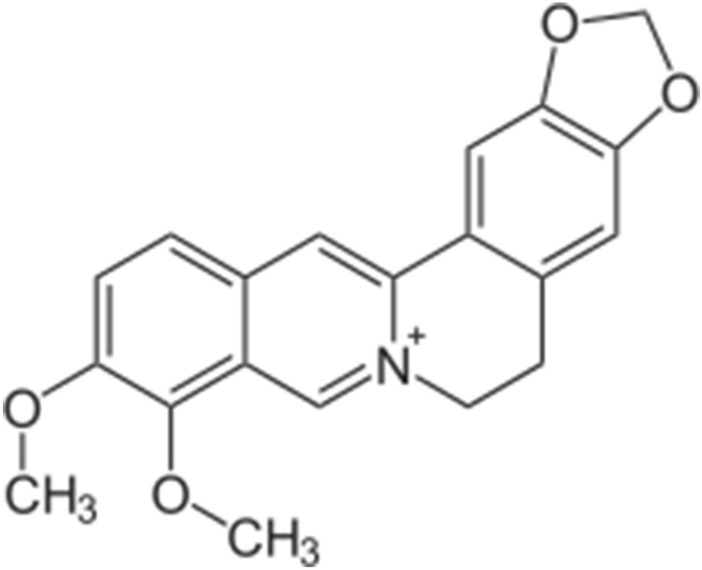
Chemical structure of berberine.

Berberine (5,6-Dihydro-9,10-dimethoxybenzo[g]-1,3-benzodioxolo[5,6-a]quinolizinium; C_20_H_18_NO_4_) (Fig. 1), a benzylisoquinoline alkaloid, is a well-known quaternary ammonium salt that is usually found in the roots of *Phellodendron amurense* and *Coptis chinensis*. Recently, Zhang et al. reported that berberine treatment significantly downregulates the expression of pro-inflammatory cytokines, such as monocyte chemotactic protein (MCP)-1, interleukin (IL)-6, and tumor necrosis factor (TNF)-α^1^. Jiang et al. reported that berberine suppresses IL-1β secretion that was induced by the activation of the NLRP3 inflammasome in macrophages^2^.

Inflammation is a necessary immunoreaction against pathogenic infection in the human body. It sometimes cause hyper-production of pro-inflammatory mediators, such as nitric oxide (NO), cytokines, chemokines, and prostaglandins. Cytokines, including chemokines and growth factors, also play an important role in hypersensitive reactions against both exogenous and endogenous antigens. Among immune cells, macrophages play a major role in defending the human body against infectious pathogens. Of course, macrophages produce many kinds of inflammatory mediators, including NO, Prostaglandin E2 (PGE2), first apoptosis signal receptor (Fas; CD95), cytokines, chemokines, growth factors, and prostaglandins, against invasive bacteria, fungi, and viruses.

In these days, severe acute respiratory syndrome coronavirus 2 (SARS‑CoV‑2) infection has caused global disruption. SARS‑CoV‑2 is thought to bring about hyper-inflammation and cytokine storm, which could be led to multiple organic failures. Thus, it is meaningful to investigate inhibitory effects of natural products on viral infection-associated inflammation. Interestingly, Kim et al. reported in 2008 that in vitro coronavirus replications are inhibited by Coptidis rhizoma and Phellodendron cortex, which are well known to contain berberine^3^.

During viral infections, many double-stranded RNAs are produced by viral replication, which causes viral inflammation. In this study, polyinosinic–polycytidylic acid (poly I:C), a synthetic analog of double-stranded RNA (dsRNA), was used to provoke a hyper-inflammatory reaction in RAW 264.7 mouse macrophages, producing various inflammatory mediators, such as NO, PGE2, Fas, granulocyte macrophage colony-stimulating factor (GM-CSF), leukemia inhibitory factor (LIF), lipopolysaccharide-induced CXC chemokine (LIX; CXCL5), chemokine ligand 5 (CCL5; RANTES), macrophage inflammatory protein (MIP)-1α, and MIP-2, as well as releasing intracellular calcium. Additionally, poly I:C increased mRNA expression levels of signal transducer and activated transcription 1 (*Stat1*), *Stat3*, C/EBP-homologous protein (*Chop*; *Ddit3*), and *Fas* in RAW 264.7 cells as well as phosphorylation of p38 mitogen-activated protein kinase (MAPK), ERK1/2, STAT3, and I-kappa-B-alpha (IkB-α).

In the previous reports, we showed that bioactive compounds, such as wogonin, oroxylin A, quercetin, and baicalein, exert anti-inflammatory effects on RAW 264.7 cells induced by poly I:C^4–6^. However, the inhibitory effect of berberine on macrophages induced by viral infection has not yet been reported. In the current study, we found that berberine has anti-inflammatory effects in poly I:C-induced RAW 264.7 cells via the endoplasmic reticulum (ER) stress-related calcium-CHOP/STAT pathway.

## Results

### Effects of berberine on cell viability

**Figure 2:**
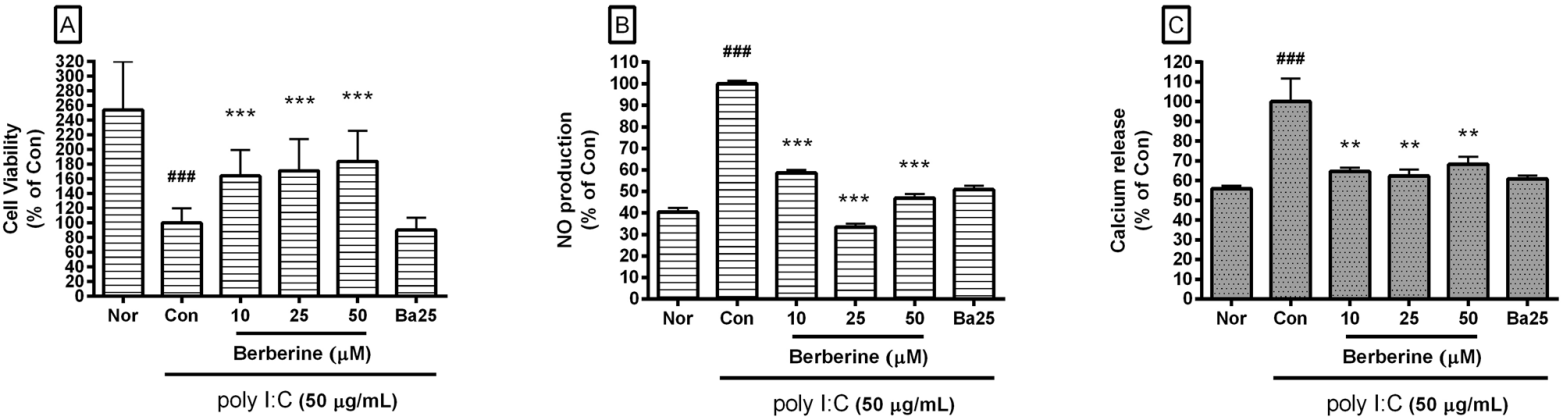
Effect of berberine on cell viability, NO production, and calcium in poly I:C-induced RAW 264.7 mouse macrophages. After 24 h incubation, cell viability (**A**) was evaluated by a modified MTT assay, and NO production (**B**) was measured by the Griess reaction assay. Calcium release (**C**) was measured with Fluo-4 calcium assay after 18 h incubation. Normal group (Nor) was treated with media only. Control group (Con) was treated with poly I:C (50 µg/mL) alone. Ba25 denote baicalein (25 µM). Values are the mean ± SD of three independent experiments. ^###^*p* < 0.001 vs. Nor; **p < 0.01 vs. Con; ***p < 0.001 vs. Con.

In this study, the cell viability in RAW 264.7 incubated with poly I:C (50 µg/mL) for 24 h was 39.37 ± 7.82% of the normal value. In the addition, cell viabilities in poly I:C-induced RAW 264.7 incubated with berberine at concentrations of 10, 25, and 50 µM were 164.17 ± 35.34%, 171.2 ± 42.97%, and 183.99 ± 41.35% of the control (poly I:C only) value (Fig. 2A). Data mean that berberine alleviates the cytotoxic effects of poly I:C on RAW 264.7. With this result, we chose berberine concentrations of up to 50 μM for subsequent experiments.

### Effects of berberine on NO production

Berberine significantly inhibited excessive production of NO in poly I:C-induced RAW 264.7 cells (Fig. 2B). The productions of NO in poly I:C-induced RAW 264.7 cells incubated with berberine at concentrations of 10, 25, and 50 µM for 24 h were 58.55 ± 1.37%, 33.49 ± 1.58%, and 46.9 ± 1.8% of the control (poly I:C only) value, respectively.

### Effect of berberine on intracellular calcium release

Berberine significantly inhibited the calcium release in poly I:C-induced RAW 264.7 cells (Fig. 2C). The calcium release in poly I:C-induced RAW 264.7 incubated with berberine at concentrations of 10, 25, and 50 µM for 18 h were 64.7 ± 1.89%, 62.35 ± 3.25%, and 68.27 ± 3.8% of the control (poly I:C only) value, respectively.

### Effect of berberine on cytokine production

**Figure 3:**
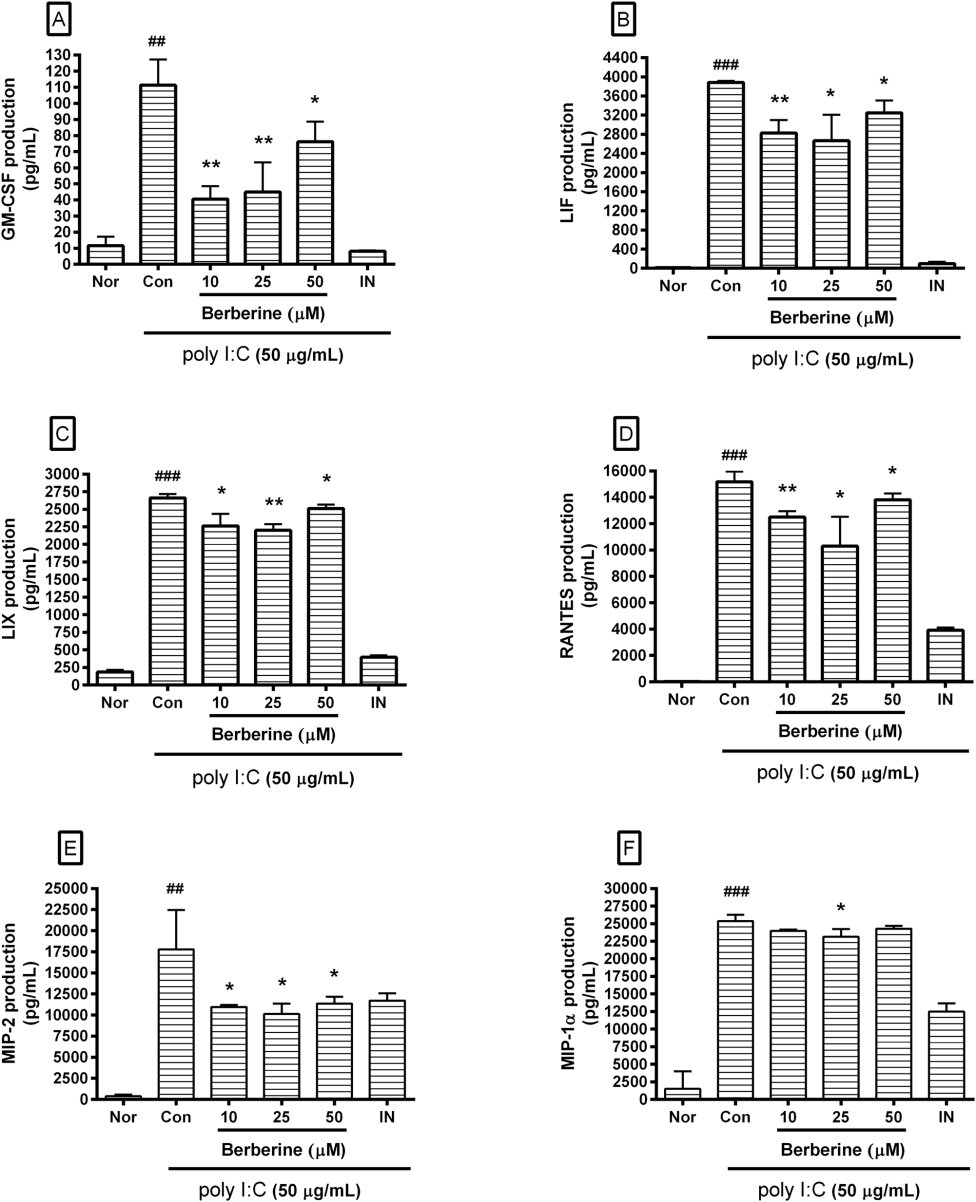
Effect of berberine on production of GM-CSF, LIF, LIX, RANTES, MIP-2, and MIP-1α in poly I:C-induced RAW 264.7 mouse macrophages. Fluorescence intensity of each cytokine in the culture medium was measured by a Multiplex-bead-based cytokine assay after 24 h incubation. Normal group (Nor) was treated with media only. Control group (Con) was treated with poly I:C (50 µg/mL) alone. IN denotes indomethacin (0.5 µM). Values are the mean ± SD of three independent experiments. ^###^*p* < 0.001 vs. Nor; *p < 0.05 vs. Con; **p < 0.01 vs. Con; ***p < 0.001 vs. Con.

Berberine decreased cytokine production in poly I:C-induced RAW 264.7 cells (Fig. 3) significantly. We found the following:

#### GM-CSF production

In RAW 264.7 incubated with media only, poly I:C only, berberine (10 µM) plus poly I:C, berberine (25 µM) plus poly I:C, berberine (50 µM) plus poly I:C, and indomethacin (0.5 µM) plus poly I:C for 24 h was 11.67 ± 5.51 pg/mL, 111.33 ± 15.95 pg/mL, 40.5 ± 8.05 pg/mL, 45 ± 18.36 pg/mL, 76.17 ± 12.55 pg/mL, and 8.17 ± 0.58 pg/mL, respectively;

#### LIF production

Was 13.83 ± 2.47 pg/mL, 3882 ± 32.22 pg/mL, 2823.5 ± 271.99 pg/mL, 2663.33 ± 544.29 pg/mL, 3247 ± 258.45 pg/mL, and 101.33 ± 36.36 pg/mL;

#### LIX production

Was 186.17 ± 30.19 pg/mL, 2662.33 ± 54.82 pg/mL, 2263.83 ± 173.57 pg/mL, 2201.83 ± 87.51 pg/mL, 2510.17 ± 58.06 pg/mL, and 396.33 ± 26.17 pg/mL;

#### RANTES production

Was 37.33 ± 18.61 pg/mL, 15,174.33 ± 791.08 pg/mL, 12,504.67 ± 444.89 pg/mL, 10,301.67 ± 2223 pg/mL, 13,804 ± 499.75 pg/mL, and 3922.167 ± 197.44 pg/mL;

#### MIP-2 production

Was 380.17 ± 218.45 pg/mL, 17,803.67 ± 4642.22 pg/mL, 10,973.88 ± 253.51 pg/mL, 10,138.25 ± 1231.07 pg/mL, 11,357.63 ± 813.8 pg/mL, and 11,719.67 ± 874.66 pg/mL;

#### MIP-1α production

Was 1518.33 ± 2465.6 pg/mL, 25,364.13 ± 908.61 pg/mL, 23,971.17 ± 185.4 pg/mL, 23,135 ± 1122.51 pg/mL, 24,299.63 ± 403.08 pg/mL, and 12,522.5 ± 1143.2 pg/mL;

These data indicated that berberine might alleviate hyper-inflammatory reaction leading to excessive production of chemokines and growth factors in macrophages stimulated by poly I:C.

### Effect of berberine on mRNA expression

**Figure 4:**
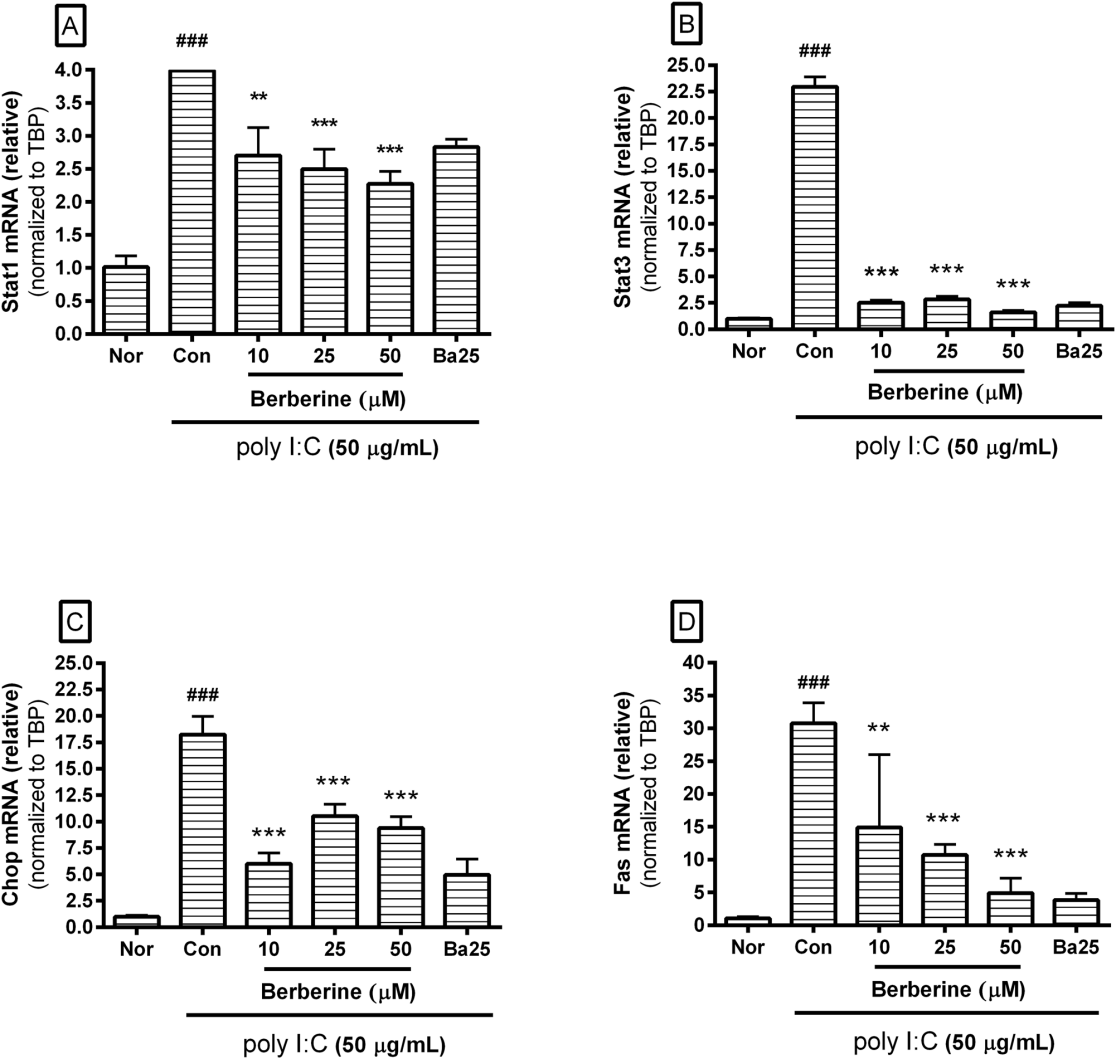
Effect of berberine on mRNA expression of *Stat1*, *Stat3*, *Chop*, and *Fas* in poly I:C-induced RAW 264.7 mouse macrophages. After 18 h incubation, the mRNA expression of *Stat1*, *Stat3*, *Chop*, and *Fas* was measured by real-time RT-PCR. The mRNA expressions were normalized to the housekeeping gene *Tbp* mRNA. Normal group (Nor) was treated with media only. Control group (Con) was treated with poly I:C (50 µg/mL) alone. Ba25 denotes baicalein (25 µM). Values are the mean ± SD of three independent experiments. ^###^*p* < 0.001 vs. Nor; **p < 0.01 vs. Con; ***p < 0.001 vs. Con.

Figure 4 shows the data on the effect of berberine on mRNA expression of *Stat1*, *Stat3*, *Chop*, and *Fas*. These results mean that berberine inhibits the inflammatory reaction in poly I:C-induced macrophages via the ER stress-related CHOP pathway. The effects were as follows: the mRNA expression of *Stat1* in poly I:C-induced RAW 264.7 incubated with berberine at concentrations of 10, 25, and 50 µM for 18 h was 67.74 ± 10.6%, 62.61 ± 7.5%, and 56.98 ± 4.81% of the group treated with poly I:C only, respectively; the mRNA expression of *Stat3* was 11.05 ± 0.99%, 12.41 ± 1.1%, and 7.06 ± 0.7, respectively; the mRNA expression of *Chop* was 32.96 ± 5.56%, 57.71 ± 6.24%, and 51.46 ± 5.9, respectively; the mRNA expression of *Fas* was 48.44 ± 36.12%, 34.87 ± 5.11%, and 15.95 ± 7.42, respectively.

### Effect of berberine on phosphorylation of p38 MAPK, ERK1/2, STAT3, and IkB-α

**Figure 5:**
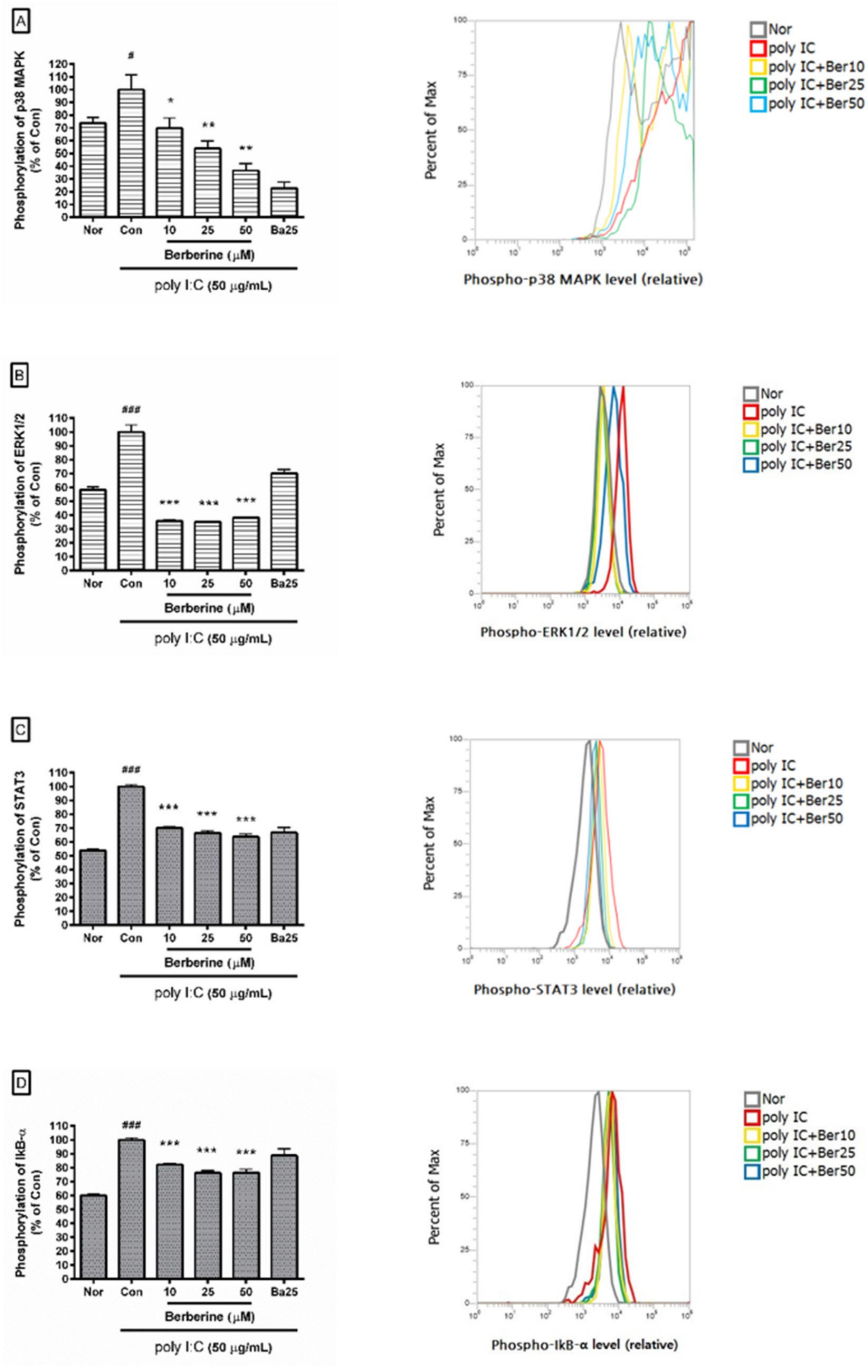
Effect of berberine on the phosphorylation of p38 MAPK (**A**), ERK 1/2 (**B**), STAT3 (**C**), and IkB-α (**D**) in poly I:C-induced RAW 264.7 cells. After 24 h incubation, the phosphorylation of p38 MAPK, ERK 1/2, STAT3, and IkB-α was measured by Flow Cytometric Analysis. Normal group (Nor) was treated with media only. Control group (Con) was treated with poly I:C (50 µg/mL) alone. Ba25 denotes baicalein (25 µM). Ber10, Ber25, and Ber50 mean 10, 25, and 25 µM of berberine, respectively. Values are the mean ± SD of three independent experiments. ^###^*p* < 0.001 vs. Nor; ^#^*p* < 0.05 vs. Nor; ***p < 0.001 vs. Con; **p < 0.01 vs. Con; *p < 0.05 vs. Con.

We found that berberine significantly inhibited the phosphorylation of p38 MAPK, ERK 1/2, STAT3, and IkB-α in poly I:C-induced RAW 264.7 cells (Fig. 5). In detail, the phosphorylation of p38 MAPK in poly I:C-induced RAW 264.7 cells incubated with berberine at concentrations of 10, 25, and 50 µM for 24 h was 69.93 ± 7.97%, 53.93 ± 5.94%, and 36.62 ± 5.29% of the control group treated with poly I:C (50 µg/mL) only, respectively; the phosphorylation of ERK1/2 was 35.87 ± 0.65%, 35.28 ± 0.18%, and 38.33 ± 0.51%; the phosphorylation of STAT3 was 70.06 ± 0.82%, 66.6 ± 1.64%, and 63.83 ± 2.05%; the phosphorylation of IkB-α was 82.25 ± 0.96%, 76.28 ± 1.88%, and 76.48 ± 2.46%. The data mean that berberine modulates the inflammatory reaction in poly I:C-induced mouse macrophages via MAPK, Nuclear factor kappa B (NF-kB), and STAT signaling.

### Effect of berberine on levels of Fas and PGE2

**Figure 6:**
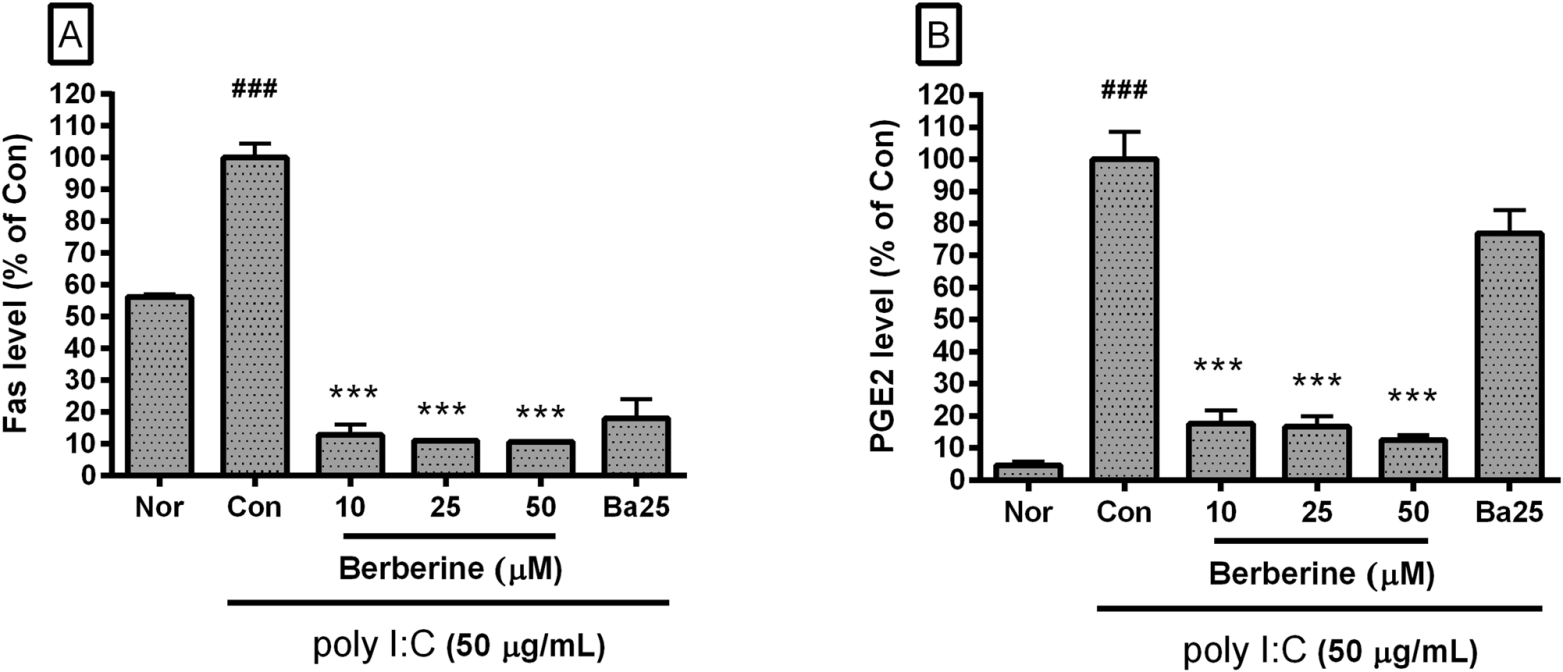
Effect of berberine on levels of Fas and Prostaglandin E2 (PGE2) in poly I:C-stimulated RAW 264.7 mouse macrophages. The levels of Fas (**A**) was evaluated by Flow Cytometric Analysis. The level of PGE2 (**B**) was measured by Prostaglandin E_2_ parameter assay. Normal group (Nor) was treated with media only. Control group (Con) was treated with poly I:C (50 µg/mL) alone. Ba25 denotes baicalein (25 µM). Values are the mean ± SD of three independent experiments. ^###^*p* < 0.001 vs. Nor; ***p < 0.001 vs. Con.

Data represented that berberine significantly decreases the levels of Fas and PGE2 in poly I:C-stimulated RAW 264.7 cells (Fig. 6). Concretely, the level of Fas in poly I:C-stimulated RAW 264.7 cells incubated with berberine at concentrations of 10, 25, and 50 µM was 12.8 ± 3.39%, 10.92 ± 0.42%, and 10.6 ± 0.35% of the control group treated with poly I:C (50 µg/mL) only, respectively; the level of PGE2 was 17.58 ± 4.2%, 16.69 ± 3.11%, and 12.41 ± 1.52%. These data mean that berberine modulates the inflammatory reaction in poly I:C-stimulated mouse macrophages via inhibiting the production of PGE2 and Fas.

## Discussion

Berberine is a well-known quaternary ammonium salt that is usually found in the roots of such plants as *Phellodendron amurense* (Amur corktree), *Berberis vulgaris* (barberry), *Berberis aristata* (tree turmeric), *Mahonia aquifolium* (Oregon grape), *Hydrastis Canadensis* (goldenseal), *Xanthorhiza simplicissima* (yellow root), *Coptis chinensis* (Chinese goldthread), *Tinospora cordifolia*, *Argemone mexicana* (prickly poppy), and *Eschscholzia californica* (Californian poppy)^7^. For pharmacological efficacy of berberine, Cicero and Baggioni reported that berberine has anti-inflammatory, antioxidant, neuroprotective, and cardiovascular protective effects, which might be used to manage chronic cardiometabolic disorders^7^. Kumar et al. reported that berberine has been used in Ayurvedic and Chinese medicine for its antimicrobial, antiprotozoal, antidiarrheal, and antitrachoma activity; it might ameliorate several disorders, including metabolic, neurological, and cardiological problems^8^. Lee et al. also reported that berberine improves memory impairment, alleviates memory-associated decreases in cholinergic immunoreactivity, restores brain-derived neurotrophic factor in the hippocampus of rats administered with scopolamine, and may improve cognitive function by stimulating cholinergic enzyme activity^9^. Actually, Lee et al. demonstrated that berberine decreases significantly the expression of IL-1β, TNF-α, and cyclooxygenase-2 mRNA in the hippocampus of rats administered with scopolamine^9^. Zhang et al. reported that berberine treatment significantly downregulates the production of pro-inflammatory cytokines and suppresses inflammatory responses in lipopolysaccharide (LPS)-stimulated macrophages through inhibition of NF-κB signaling^1^. Jiang et al. reported that berberine suppresses IL-1β secretion that was induced by the activation of the NLRP3 inflammasome in macrophages, which could be an important therapeutic target in atherosclerosis^2^.

Immunity is important for human health. Inflammation is necessary in immune activity. Inflammatory mediators, such as NO, prostaglandins, and cytokines, play a significant role in immune reactions and inflammatory phenomena. Pro-inflammatory mediators play an important role in removing pathogens such as bacteria, fungus, and virus. Sometimes an unregulated infection might cause a syndrome of excessive cytokine production, i.e., “cytokine storm (hypercytokinemia)”^10–14^.

Viral infection as well as bacterial infection might evoke massive production of pro-inflammatory mediators. In spite of modern developments in drugs and vaccines, there are still many intractable disorders caused by pathogens. Thus, it is reasonable to search for a material to modulate viral inflammation and virus-induced hypercytokinemia.

For reference, we previously reported how immuno-inflammatory activities of various natural products may affect macrophages^15–21^. For viral inflammation, we showed that flavonoids, such as quercetin, oroxylin A, and baicalein, exert anti-inflammatory effects on RAW 264.7 cells induced by poly I:C^4–6^.

In these studies, poly I:C was used to bring about inflammatory reactions in mouse macrophages, which are a virus-induced cellular inflammation model. It is well known that dsRNA is produced in immune cell through viral replication and elicit cytotoxic responses^22^. In the current study, our data show that berberine alleviates the cytotoxic effects of poly I:C on RAW 264.7. Thus, berberine might be an inhibitor of apoptosis in macrophages stimulated by poly I:C.

In 2001, Alexopoulou et al. reported that mammalian toll-like receptor 3 could recognize dsRNA and gradually induce cytokine production via NF-kB and MAPK activation^23^. This study shows that berberine significantly inhibited the production of NO, PGE2, Fas, GM-CSF, LIF, LIX, RANTES, and MIP-2 as well as calcium release and phosphorylation of p38 MAPK, ERK1/2, IkB-α, and STAT3 in poly I:C-stimulated RAW 264.7 cells. These results suggest that the anti-inflammatory effect of berberine in poly I:C-stimulated RAW 264.7 cells might involve the calcium signaling pathway, including activation of MAPK, NF-kB, and STAT3.

It is not yet fully explained how viral infection causes an ER stress with intracellular calcium release and CHOP expression. In relation to CHOP signaling pathway in stressed cells, Wang and Ron reported in 1996 that CHOP is phosphorylated and activated by p38 MAPK in stressed cells under conditions of stress^24^. In 2006, Endo et al. reported that the start of LPS-induced inflammation cascade in lung might be triggered through ER stress-CHOP pathway including the activation p38 MAPK and NF-kB^25^. With CHOP-induced apoptosis in macrophages, Gotoh et al. reported in 2004 that ER stress-CHOP pathway is involved in NO-mediated apoptosis in LPS plus interferon-γ-stimulated macrophages^26^. Although NO is known to be necessary for various physiological processes, it means that NO might be a crucial key factor in ER stress-CHOP pathway in virus-stimulated macrophages. Moreover, Mory M. reported meaningfully in 2007 that NO depletes ER Ca2^+^, which consequently causes ER stress and leads apoptosis in macrophages^27^. An increase in cytosolic calcium is well known to activate CAMKII, which induces the apoptotic signaling cascade in macrophages including Fas induction and activation of STAT1^28–29^.

Our experimental results show that berberine inhibits the overexpression of *Chop*, *Stat1*, *Stat3*, and *Fas* in poly I:C-stimulated RAW 264.7 cells. Hence berberine may be able to alleviate ER stress induced by dsRNA via the calcium-STAT pathway including activation of MAPK and NF-kB. But this study could not evaluate whether calcium signaling causes CAMKII activation in poly I:C-stimulated RAW 264.7 cells or not. These studies also could not verify exactly the effect of berberine on Type I interferons signaling in poly I:C-stimulated RAW 264.7 cells. It is meaningful that intracellular calcium is an important signaling molecule of ER stress and increases with *Chop* overexpression in poly I:C-stimulated RAW 264.7 cells.

Finally, our data mean that berberine exerts an anti-inflammatory effect related to its inhibition of NO, PGE2, Fas, GM-CSF, LIF, LIX, RANTES, and MIP-2 in poly I:C-stimulated RAW 264.7 cells via the ER stress-related calcium-CHOP/STAT pathway. The actual effect of berberine on viral inflammation deserves to be further studied.

## Materials and methods

We purchased DMEM, FBS, penicillin, streptomycin, PBS, and other tissue-culture reagents from Thermo Fisher Scientific (Waltham, MA, USA). Poly I:C, berberine, baicalein, indomethacin, Griess reagent, and all other chemicals for cell culture from Sigma-Aldrich (St. Louis, MO, USA). Multiplex cytokine assay kits from Millipore (Billerica, MA, USA); Fluo-4 calcium assay kits from Molecular Probes (Eugene, OR, USA); Real-time RT-PCR kits from Bio-Rad (Hercules, CA, USA); Prostaglandin E_2_ parameter assay kit from R&D Systems (Minneapolis, MN, USA). Phospho-p38 MAPK Antibody (T180/Y182) (eBioscience 17-9078-42), Phospho-ERK1/2 Antibody (Thr202, Tyr204) (eBioscience 12-9109-42), phospho-STAT3 Antibody (Tyr705) (eBioscience 12-9033-42), phospho-IkB-α Antibody (Ser32, Ser36) (eBioscience 12-9035-42), and CD95 Antibody (APO-1/Fas) (eBioscience 12-0951-83), Mouse IgG1 kappa Isotype Control (eBioscience 12-4714-81), and Mouse IgG2b kappa Isotype Control (eBioscience 12-4732-81) from Life Technologies Corporation (Carlsbad, CA, USA). All other solutions for flow cytometric analysis from Thermo Fisher Scientific.

### Cell viability

RAW 264.7 cells were obtained from the Korea Cell Line Bank (Seoul, Korea). Cells were cultured in DMEM supplemented with 10% FBS containing 100 U/mL of penicillin and 100 µg/mL of streptomycin at 37 °C in a 5% CO_2_ humidified incubator. RAW 264.7 were cultured for 24 h, and cell viability was evaluated with the tetrazolium-based colorimetric assay (MTT) according to the previous study with a microplate reader (Bio-Rad)^4–6^.

### NO concentration

After 24 h incubation, NO concentration in culture medium was measured by the Griess reaction as in previous studies with a microplate reader (Bio-Rad)^4–6^.

### Intracellular calcium assay

Fluo-4 AM is a well-known fluorescent Ca^2+^ indicator used for the in-cell measurement of calcium signaling. After 18 h of treatment in 96-well plates, the intracellular calcium signaling from each well containing RAW 264.7 (1 × 10^5^ cells/well) was identified using Fluo-4 NW Calcium Assay Kits (Thermo Fisher Scientific) following the protocol of our previous study^4–6^. Specifically, after incubating the cells with poly I:C and/or berberine for 18 h at 37 °C, we measured the intracellular calcium level using Fluo-4 assay with a spectrofluorometer (Dynex, West Sussex, UK) with excitation and emission filters of 485 nm and 535 nm, respectively.

### Multiplex cytokine assay

We did this assay with multiplex cytokine assay kits and a Bio-Plex 200 suspension array system (Bio-Rad) as described previously^4–6^. After 24 h incubation, the following cytokine productions were analyzed: GM-CSF; LIF; LIX; RANTES; MIP-2; MIP-1α.

### Real-Time RT-PCR assay

In 6-well plates, RAW 264.7 (3 × 10^5^ cells/well) were incubated in each well with or without berberine in poly I:C for 18 h. After 18 h incubation, total RNA of each well was isolated using NucleoSpin RNA kit (Macherey–Nagel, Duren, Germany). Then, cDNA of the RNA samples was produced using iScript cDNA Synthesis kit (Bio-Rad). The mRNA expression of *Stat1*, *Stat3*, *Chop*, and *Fas* were evaluated with real-time RT-PCR using an Experion Automatic Electrophoresis System (Bio-Rad) and Bio-Rad CFX 96 Real-Time PCR Detection System (Bio-Rad)^6^. *Tbp* was used for RNA normalization. The sequence of each primer set is showed in Table 1.

**Table 1:** Primers used for RT-PCR analysis.

Name^a^	Forward primer (5′–3′)	Reverse primer (5′–3′)
*Stat1*	TGAGATGTCCCGGATAGTGG	CGCCAGAGAGAAATTCGTGT
*Stat3*	GTCTGCAGAGT TCAAGCACCT	TCCTCAGTCACGATCAAGGAG
*Chop*	CCACCACACCTGAAAGCAG	TCCTCATACCAGGCTTCCA
*Fas*	CGCTGTTTTCCCTTGCTG	CCTTGAGTATGAACTCTTAACTGTGAG
*Tbp*	GGGGAGCTGTGATGTGAAGT	CCAGGAAATAATTCTGGCTCA

^a^Primer’s names; signal transducers and activators of transcription 1 (*Stat1*), *Stat3*, C/EBP homologous protein (*Chop*), first apoptosis signal receptor (*Fas*), TATA box binding protein (*Tbp*).

### Flow cytometric analysis

In 6-well plates, RAW 264.7 (3 × 10^5^ cells/well) were incubated in each well with or without berberine in poly I:C for 24 h. After 24 h incubation, cells were harvested and washed with Flow Cytometry Staining Buffer (Thermo Fisher Scientific). Cells were stained with antibodies for phospho-p38 MAPK, phospho-ERK1/2, phospho-STAT3, phospho-IkB-α, and Fas according to the manufacture’s protocol as described previously^21^. Prior to antibody staining, cells were fixed and permeabilized using Fix Buffer I (Thermo Fisher Scientific) and Perm Buffer III (Thermo Fisher Scientific), respectively. Stained cells were analyzed on the Attune NxT flow cytometer (Thermo Fisher Scientific). Data were obtained from the mean fluorescent intensities of samples. Details for startup, proper calibration and operation of the Attune can be found in the Attune User Guide (https://assets.thermofisher.com/TFS-Assets/LSG/manuals/100024235_AttuneNxT_HW_UG.pdf). A serial gating strategy used forward scatter versus side scatter plots and the target antibody expression plots. Unstained cells were used as the negative controls for gating. Mouse IgG1 kappa Isotype Control was used to confirm the specificity of phospho-p38 MAPK Antibody and Fas Antibody. Mouse IgG2b kappa Isotype Control was used to confirm the specificity of phospho-ERK1/2 Antibody, phospho-STAT3 Antibody, and phospho-IkB-α Antibody. For analysis of raw data, we used Attune NxT software (Thermo Fisher Scientific). Baicalein (25 µM) was used as a positive control.

### Prostaglandin E2 assay

In 96-well plates, RAW 264.7 (1 × 10^4^ cells/well) were incubated in each well with or without berberine in poly I:C for 24 h. After 24 h incubation, the production of PGE2 in each well containing RAW 264.7 cells was evaluated using Prostaglandin E_2_ parameter assay kit (R&D Systems) according to the manufacture’s protocol. The absorbance of the end product was measured at a wavelength of 450 nm using a microplate reader (Bio-Rad)^30^.

### Statistical analysis

We present data using the mean ± SD of three independent experiments. Significant differences were examined using a one-way analysis of variance test, followed by Tukey’s multiple-comparison test with SPSS 11.0 software (SPSS, Inc., Chicago, IL, USA). In all cases, a *p* < 0.05 was considered significant.
